# A Data Augmentation-Based Evaluation System for Regional Direct Economic Losses of Storm Surge Disasters

**DOI:** 10.3390/ijerph18062918

**Published:** 2021-03-12

**Authors:** Hai Sun, Jin Wang, Wentao Ye

**Affiliations:** 1 College of Engineering, Ocean University of China, Qingdao 266100, China; wj7699@stu.ouc.edu.cn; 2 Institute of Marine Development of the Ocean University of China, Ocean University of China, Qingdao 266100, China; 3 School of Computer Science and Engineering, University of Electronic Science and Technology of China, Chengdu 611731, China; 2017070903015@std.uestc.edu.cn

**Keywords:** economic losses, storm surge, XGBoost, data augmentation, KNN-GN

## Abstract

The accurate prediction of storm surge disasters’ direct economic losses plays a positive role in providing critical support for disaster prevention decision-making and management. Previous researches on storm surge disaster loss assessment did not pay much attention to the overfitting phenomenon caused by the data scarcity and the excessive model complexity. To solve these problems, this paper puts forward a new evaluation system for forecasting the regional direct economic loss of storm surge disasters, consisting of three parts. First of all, a comprehensive assessment index system was established by considering the storm surge disasters’ formation mechanism and the corresponding risk management theory. Secondly, a novel data augmentation technique, k-nearest neighbor-Gaussian noise (KNN-GN), was presented to overcome data scarcity. Thirdly, an ensemble learning algorithm XGBoost as a regression model was utilized to optimize the results and produce the final forecasting results. To verify the best-combined model, KNN-GN-based XGBoost, we conducted cross-contrast experiments with several data augmentation techniques and some widely-used ensemble learning models. Meanwhile, the traditional prediction models are used as baselines to the optimized forecasting system. The experimental results show that the KNN-GN-based XGBoost model provides more precise predictions than the traditional models, with a 64.1% average improvement in the mean absolute percentage error (MAPE) measurement. It could be noted that the proposed evaluation system can be extended and applied to the geography-related field as well.

## 1. Introduction

When a typhoon makes landfall, strong winds, low pressures, and high waves are generated. The wind stress and the low pressure at the typhoon center will cause the sea level to rise. High waves will result in an abnormal increase in tides. The combination of abnormal and normal tides will form a typhoon storm surge, which will increase the average water level from one meter to more than five meters. In 2019, the super typhoon 1909 “Lichma” struck the southeast coast of China, causing eight provinces to suffer severe storm surge disasters. The maximum storm surge caused by it was as high as 312 cm, and the direct economic loss was nearly CNY 10.3 billion (approximately USD 1.6 billion) [1]. The significant impact of the typhoon storm surge on economic and social development has drawn attention from scholars. Disaster economic loss assessment could significantly support the disaster prevention decision and management of typhoon storm surge. Therefore, the research on how to quickly and accurately forecast storm surge disasters’ economic loss is vital and meaningful.

At first, many researchers preferred to use the statistical regression model, mostly focusing on the relationship between a few indicators and disaster losses, to forecast disaster losses caused by typhoon storm surges. Schmidt et al. [2] utilized the polynomial regression equations to reflect the relation between no more than two hazard factors and disaster losses. After that, to improve the prediction’s accuracy, the researchers made improvements in two aspects: by using more complex models and increasing the number of hazard factors. Murnane and Elsner [3] applied a quantile regression to explore the relationship between meteorological indicators and hurricane losses. Kim et al. [4] and Qi et al. [5] increased the number of hazard factors to seven and nine, respectively, in assessing typhoon storm surge hazard risk by different multivariate analysis methods. These models achieved better performance. Besides, several econometric models have been used by some researchers to assess the disaster losses, such as computable general equilibrium models and input–output models [6]. These statistical models have achieved specific results in solving weakly non-linear problems of low dimensionality. However, disasters are complex, high-dimensional, and strongly non-linear systems, which are still not well predicted by these models. Therefore, an effective economic loss assessment model for natural disasters should include as many hazard factors as possible and reflect the complicated non-linear relationship between hazards and direct economic losses.

Machine learning has recently become popular in natural disaster research [7]. Machine learning algorithms improve prediction accuracy by building more extensive and complex learning networks to fit the complex non-linear relationships between multi-dimensional variables. Hence, more and more scholars have tried to apply machine learning algorithms to the typhoon storm surge’s economic loss assessment. Lou et al. [8] selected 23 disaster-causing factors from four dimensions as input data to construct a loss assessment model of tropical cyclone disasters based on support vector regression (SVR). Wang et al. [9] and Yuan et al. [10] built an evaluation index system and utilized the backpropagation neural network (BPNN) model to forecast the storm surge’s economic damage. They optimized the BPNN with the beetle antennae search (BAS) algorithm and the Levenberg Marquardt (LM) algorithm, respectively, to improve the prediction accuracy. Besides, Lin et al. [11] used vector space model (VSM) to correct the result of BPNN to make a more precise prediction. Chen et al. [12] combined three models, GA (genetic algorithms)–Elman neural networks, SVR, and generalized regression neural networks (GRNN), to predict the tropical cyclone disaster loss. This combined model is like a simple “stacking” model in the ensemble learning, which made progress in the prediction performance.

However, the previous works have drawbacks in two aspects: the regression model’s generalization ability and the data scarcity. For the former, although various machine learning models are employed for natural disasters [7], they are almost all related to BPNN and SVR in the field of economic loss forecasting. Ensemble learning is a widely-used algorithm [13,14,15,16] that combines several machine learning techniques into an ensemble model to reduce deviation and improve prediction accuracy [17]. Zhao et al. [18] used an ensemble learning model Adaboost-BPNN for forecasting direct economic losses of marine disasters. Besides, ensemble learning is rarely used in this field. Thereby, we introduced XGBoost [19] into the field of direct economic loss evaluation caused by storm surge disasters. For the latter one, without big data, machine learning methods are prone to overfitting, which means that the model perfectly fits the training dataset but is not generalized well to unknown data [20]. However, it is unrealistic and impossible to obtain large amounts of storm surge data from the historical literature due to the limited number of storm surges in practice. Zhao et al. [18] adapted four interpolation methods to tackle the small sample issue, but the interpolation methods in this article cannot reflect the disaster processes’ randomness. Besides, few studies are focusing on the data scarcity problem in this scope. Inspired by the standard methods for solving data scarcity in the field of deep learning, we concentrate on data augmentation [21], a technique that could not only enhance the size and quality of training data to reduce overfitting but also induce randomness. To solve the overfitting problem caused by data scarcity, we propose a novel data augmentation technique named the k-nearest neighbor-Gaussian noise (KNN-GN) algorithm.

The main findings of this research can be concluded in three aspects. Firstly, we established a comprehensive assessment index system for the regional direct economic loss evaluation of storm surge disasters. Secondly, we put forward a novel data augmentation technique, k-nearest neighbor-Gaussian noise (KNN-GN), to settle the data scarcity problem. KNN-GN expands the samples by injecting random directional noise, making the augmented sample more satisfactory for the ensemble model. Thirdly, we conducted an experiment to explore the most optimized ensemble learning model, named KNN-GN-based XGBoost. To verify the proposed method’s prediction effectiveness, we compared the model to other traditional ones, such as BPNN and SVR, based on the mean absolute percentage error (MAPE), a commonly used performance metric. The damage assessment model proposed in this paper could provide a quick and accurate estimation of direct economic losses shortly after a storm surges disaster occurs, just with some investigated and available disaster loss information. This rapid post-disaster prediction technology may help organizations to make better disaster prevention decisions to avoid the more significant impact of disasters.

## 2. Methodology

The evaluation system consists of two parts: (1) a comprehensive index system of storm surge disasters and (2) a KNN-GN-based XGBoost regression model. In the following subsections, the two parts will be described in detail.

### 2.1. The Disaster Loss Assessment Index System

A reasonable assessment index system of storm surge disaster loss should fully consider the formation mechanism of storm surge disasters, disaster system theory, and risk management theory. The storm surge disasters will occur in the wake of high storm surge levels. Then, the seawater will pass over the dam and flood the farmland, production, and living facilities, which will cause certain economic losses. Multiplied factors affect economic losses. To better learn the relationship between factors and natural disaster loss or risk, numerous researchers developed the index system-based assessment method. Some of them established the index system considering both biophysical and anthropogenic factors to assess the disasters losses and risk in China [9,22,23,24,25], Greece [26], the Netherlands [27], the United States [28,29,30], Brazil [31], Pakistan [32], and other regions.

Assessment indicators need to be accurate, specific, and quantifiable. Qualitative indicators should also be quantified as much as possible to avoid a greater degree of subjectivity. At the same time, various hazard-causing factors should be fully considered to form a relatively complete assessment index system. The selection of indicators must be relatively easy to obtain at this stage, and can be obtained through investigations or experiments. Only in this way can the indicator system be valid and objective. Based on the above principles and referring to 17 representative articles in the past twenty-one years from 2000 to 2020, we considered the intensity of the storm surge disaster hazard, the natural environment, and the socio-economic development of the affected area to propose a comprehensive evaluation index system from four primary criteria: disaster-causing factors, disaster-formative environment, hazard-bearing bodies, and disaster prevention capabilities. Based on the four primary criteria, the loss assessment index system, including 16 indicators, is constructed, as shown in Table 1, considering the principles of objectivity, accessibility, integrity, and low correlation. These 16 indicators have a direct or indirect influence on the regional direct economic loss and are relatively easy to obtain. In the experiment, we choose these 16 indicators as the features in the regression models.

#### 2.1.1. Disaster-Causing Factors

This part includes three indicators: maximum storm surge (cm), exceeding the local warning water level (cm), and typhoon duration (h). Storm surge disasters are generated by the abnormal rise and fall of seawater caused by severe atmospheric disturbance. The maximum water increase and tide level are the direct signs of the seawater changes after the arrival of the storm surge, which can directly reflect the intensity of the storm surge. The typhoon’s duration is the typhoon’s staying time in the study area that triggered the storm surge. This indicator reflects the impact of the typhoon on disaster areas directly and the storm surge’s intensity indirectly. Those disaster-causing factors which represent the intensity of storm surges are often used to predict the economic losses caused by storm surges, showing that three indicators in the disaster-causing factors are significantly correlated with the regional direct economic loss.

#### 2.1.2. Disaster-Formative Environment

This part mainly studies the exposure degree of the natural and social attributes in the study area to storm surge disasters. Therefore, the indicators consider three dimensions: natural environment, economic development, and population structure.

From the natural environment perspective, the indicator of the urban green area (hm^2^) was selected. It is the fundamental element for maintaining the urban ecological environment [38], helping a coastal city minimize natural disaster risks [39]. The urban green area reflects an area’s ecosystem health, and a healthy ecosystem can help to withstand storm surges effectively. Meanwhile, the green belt can effectively prevent road surface water and further reduce the economic losses caused by storm surge disasters. So, the green plant plays a vital role in social, ecological, and economic recovery after disasters such as hurricanes [40,41].

As for economic development, the aquaculture area (hm^2^) and crop sown area (hm^2^) were chosen as two indicators. The aquaculture area reflects the scale of mariculture in the disaster-affected area and the degree of dependence on the ocean. When a tidal disaster occurs, the farmland will be flooded by the seawater’s floodplain, which results in direct economic losses. The sown area of crops represents the agricultural development situation in the disaster-affected area. When a storm surge occurs, fisheries and agriculture are the most vulnerable economic entities to be affected. The larger the storm surge, the more likely it is to cause more economic losses.

The number of casualties is a part of the direct economic losses caused by storm surge disasters. Considering the population structure, we selected the proportion of the old and young population and the urban population’s proportion as two indicators. After a natural disaster, different groups of people will face different degrees of losses. Vulnerable groups and low-income families in society are often more likely to be exposed to natural disasters and find it harder to recover [42]. The first indicator adopts the proportion of children under 15 and seniors over 65 in the study area’s total population. Since children and seniors are in a relatively disadvantaged position in society, their self-rescue ability to face disasters is weaker than that of young people [43]. When the proportion of the old and young population in the affected areas becomes more extensive, the probability of death from injuries will also increase, impacting economic losses. Simultaneously, the overall economic development level in rural areas is not as good as that in cities because there are many low-income families in the countryside. The low housing and infrastructure conditions have made them more vulnerable to storm surge damage and difficult to recover from the disaster. The urban population’s proportion reflects the study area’s urbanization level and indirectly reflects the ratio of the population vulnerable to disasters. The higher this indicator, the less vulnerable the study area is to storm surge hazards.

#### 2.1.3. Disaster-Affected Bodies

This index shows the vulnerability of the disaster-affected bodies. The biophysical vulnerability refers to the ultimate impact of the disaster, which is usually expressed by a certain amount of losses of the system at risk [44]. Numerical simulation studies of natural hazards are now proliferating, which allows us to estimate the potential damage to a disaster more accurately by computer [45]. In the numerical simulation of storm surge hazards, the affected population and the damage to marine engineering are relatively straightforward. These two indicators are better able to help us to predict the regional direct economic losses more quickly and precisely. Hence, this paper takes the loss of the disaster area caused by the storm surge as the representation of vulnerability of the disaster body, which is characterized by two indicators: the disaster-affected population and the length of marine engineering damage (km). These two indicators can directly reflect the economic losses caused by storm surge disasters. When a place suffers a storm surge disaster, the affected population and the length of marine engineering damage are the most common and easily collected items while counting property through the survey.

#### 2.1.4. Disaster Prevention Capabilities

Disaster prevention capabilities show the research area’s ability to resist disasters, which plays a vital role in reducing the impact of disasters. Disaster prevention capabilities can reduce the economic losses and help the affected areas quickly resume production. This paper selects various indicators that characterize the research area’s disaster prevention and mitigation capabilities from two dimensions: economic development and social security.

Per capita GDP is an indicator that directly reflects the regional economic development level, as the economy is the foundation of the overall development. Good economic development can drive the growth of other aspects of society and improve disaster resilience as well. Meanwhile, the unemployment rate can reflect the region’s overall economic conditions and represent the stability of society to a certain extent. Generally speaking, regions of economic prosperity and social stability are more able to withstand natural disasters than less developed regions.

Besides, fiscal expenditure stands for the local government’s investment in various public undertakings, including facilities construction, public safety, social security, etc. The higher the index, the better the government’s ability to deal with natural disasters. Storm surge disasters not only cause direct economic losses but also often lead to casualties. Therefore, the victims’ life safety cannot be guaranteed without the support of the medical system. The number of beds per thousand people and the number of medical institutions can fully indicate the study area’s medical conditions. The higher the indicators, the stronger the rescue capability and the smaller the disaster loss. Lastly, commercial insurance costs directly show the anti-risk level of a region. Insurance is an effective means of risk transfer; thus, after a storm surge disaster, insurance can recoup parts of the regional direct economic loss and improve the entire region’s disaster resistance.

### 2.2. Data Input

#### 2.2.1. Study Area

The southeastern coast of China is one of the most severely affected areas in the world by storm surge disasters. About one-third of the world’s typhoons originate in the Northwest Pacific [46], and the southeastern coast of China is located on the main moving path of typhoons in the Northwest Pacific. Therefore, certain geographical factors have caused China’s southeastern coast to suffer typhoon storm surges for the long term. According to statistics from the *China Marine Disaster Bulletin* (2001–2019) [1], in the past 20 years, storm surge disasters have caused CNY 212.511 billion (approximately USD 32 billion) direct economic losses to the Chinese mainland.

**Figure 1:**
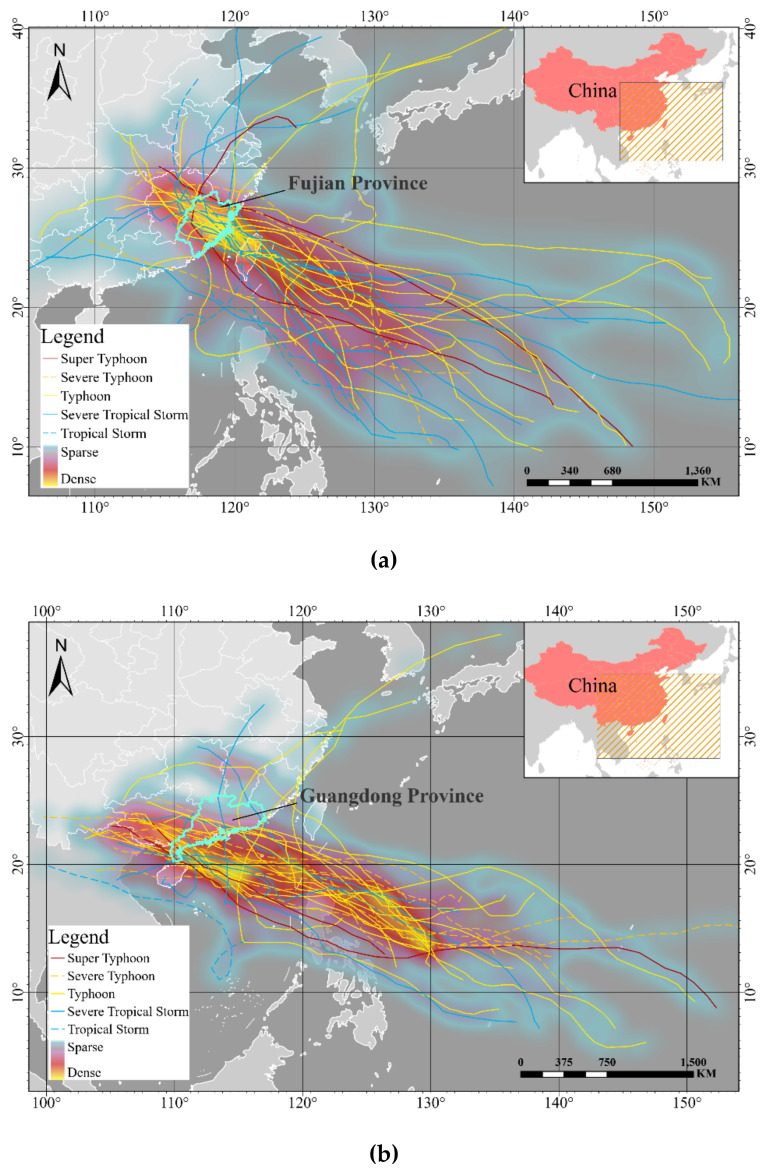
This is the landing track of selected typhoon storms. (**a**) The landing track of 32 typhoon storms affecting Fujian; (**b**) the landing track of 35 typhoon storms affecting Guangdong.

The study area in this paper is Fujian Province (115° 50′~120°40′ E, 23°30′~28°22′ N), located on the southeastern coast of China. As a typical coastal province, Fujian has a long coastline (3752 km) with low-lying coastal areas. Moreover, as one of China’s developed regions, it has a large population and a prosperous economy. The reasons that cause Fujian long-suffering from typhoon storm surges and enormous economic losses are both factors on particular geography and social economy. In the past five years, the typhoon storm surge disasters have caused the direct financial loss of Fujian about CNY 5.96 billion (about USD 0.9 billion), accounting for 99% of all marine disasters. Therefore, this paper selected 32 typhoon storms with comprehensively complete records that affected Fujian from 1995 to 2019 (as shown in Figure 1a). Besides, to evaluate the robustness of the research method, this paper also chooses 35 typhoon storms data (see in Figure 1b) of Guangdong province (109°39′~117°19′ E, 20°13′~25°31′ N), another typical coastal province in China, as the input of the robustness experiment. The data used to draw the landing track in Figure 1 is obtained from the China Meteorological Administration Tropical Cyclone Best Track Data Center (tcdata.typhoon.org.cn, accessed on 4 March, 2021) and refers to the work of Ying et al. [47].

#### 2.2.2. Data Collection

This research involves 17 variables: 16 independent variables in the index system and a dependent variable, the direct economic loss of a regional storm surge disaster. In this paper, we respectively select thirty-two and thirty-five storm surge disasters with relatively complete records in the Fujian and Guangdong provinces during the 25 years from 1995 to 2019 as research materials. The samples in Fujian are used for the main experiments, while the samples in Guangdong are used for the robustness experiment. These disaster-related data come from the following eight sources.

- *China Marine Disaster Bulletin* [1]: The Ministry of Natural Resources of China publishes this annual report on its official website to record the information of marine disasters suffered across China in the previous year. In this paper, the data of the maximum storm surge, exceeding the local warning water level, disaster-affected population, marine engineering damage length, and regional direct economic loss were collected from twenty-five bulletins from 1995 to 2019. What needs special explanation is that the regional direct economic loss data were collected, counted, and checked by local governments. The public officials classified and counted kinds of lost property caused by the disaster through the field survey, and then calculated the corresponding value to obtain the overall direct economic loss data.
- *Fujian Marine Disaster Bulletin* [48]: The Bureau of Ocean and Fisheries of Fujian Province, from 2011, offers these annual reports on its official website. Like *China Marine Disaster Bulletin,* it records the information of marine disasters but only includes data from Fujian in the previous year. Moreover, *Fujian Marine Disaster Bulletin* has supplementary records of the affected population and extra records the length of marine engineering damage in Fujian.
- *Guangdong Marine Disaster Bulletin* [49]: The Department of Natural Resources of Guangdong Province publishes these annual reports from 2013. It also offers the data of the affected population, which is not in the *China Marine Disaster Bulletin,* and the length of marine engineering damage.
- *Collection of Storm Surge Disasters Historical Data in China 1949–2009* [50]: This book is one of the achievements of a special survey program organized by the State Oceanic Administration of China. It collects and collates the detailed information of the maximum storm surge and exceeding the local warning water level of 209 typhoon storm surge disasters in China from 1949 to 2009. Because marine disaster bulletins mentioned above do not record the data of the maximum storm surge and exceeding the local warning water level of a few storm surges before 2009, we referred to this book for the supplements.
- *Central Meteorological Observatory Typhoon Website* [51]: This website displays lots of typhoon tracking information, which can be used to calculate the land impact time.
- *National Statistics of China* [52]: This is a database provided by the National Bureau of Statistics, which includes the basic natural environment and socio-economic data of each province. Eleven indicators of disaster-formative environments and disaster prevention capabilities are mainly from this database.
- *Statistical Yearbook of Fujian* [53]: This annual report is published by the Fujian Bureau of Statistics from 2000. It supplements the missing data in the *National Statistics of China* in terms of 11 indicators of disaster-formative environments and disaster prevention capabilities that are missing.
- *Statistical Yearbook of Guangdong* [54]: These annual reports also log the local socio-economic development, which are published by the Guangdong Bureau of Statistics from 2000. Similar to the *Statistical Yearbook of Fujian*, it fills in the part of missing data of 11 indicators belonging to disaster-formative environments and disaster prevention capabilities.

The above data are taken from the official website of China. To provide follow-up researchers with quicker access to the data, we have collated and uploaded the bulletin and yearbook documents [55] used in this paper for reference. What needs particular explanation is that the social capital shifts to the coastal cities as the economy develops. Greater direct economic losses occur once suffering the storm surge.

#### 2.2.3. Data Preprocessing

Step 1: missing data processing: As mentioned above, all data are from online or offline materials, including official reports and reference books. We collected as much data as possible, but there were still five missing data and three uncertain data among the thousands of data obtained. Although data augmentation techniques could fill the data scarcity, we still hope to fill these gaps through manual intervention because this provides samples with more practical information to enhance the model’s predictive effect. To make the sample data more complete and usable, we carried out preprocessing work on these rare missing data, which consisted of filling in the missing data and dealing with the uncertain data.

We filled in the data gaps in the following ways. First of all, the storm “9608Herb” that occurred in 1996 had three missing indicators, namely, the proportion of the old and young population $X_{7}$, the proportion of the urban population $X_{8}$, and commercial insurance costs $X_{16}$, because these indicators were not counted in *the Statistical Yearbook of Fujian* [53] before the year 2000. After checking the correlation between these indicators and the year, we used generalized linear regression [56] with independent variable years to fill these missing data. Secondly, the affected population and the length of marine engineering damage of the storm “0010bilis” that occurred in 2000 were completed by a kind of single imputation (set to zero) [57] due to its non-obvious regularity.

In addition, we have only three uncertain data in three indicators, namely, the maximum storm surge, exceeding the local warning water level and regional direct economic loss. The reason for the missing data is due to all uncertain data appearing in the early records. The uncertain, direct economic damage value comes from the storm “0604 Bilis”, which occurred in 2006. Because the two official reports mentioned above were investigated at different times, there are two different regional direct economic losses. Besides, due to the different observation positions where the survey crew measured the maximum storm surge and exceeding the local warning water level, there are also a few different values. It is worth noting that the gap between these different values is very small. These uncertain data could be filled by the average value of different resources.

Step 2: normalization: Every sample in this original dataset has 16 different features, each representing a different meaning of storm surge or information about the local province. Each component has a specific magnitude. For example, the mean of the feature “exceeding the local warning water level” is 155.77, while the mean of the feature “maximum storm surge” is only 30.65. To eliminate the dimensional influence between indicators, we use the normalization process to solve data indicators’ comparability. Considering that money has time value, we discount the monetary features into the present value before normalizing. Finally, normalization is generally carried out by subtracting the mean and then divided by the standard deviation of given data. The formula is defined as follows:

(1) $$y=\frac{x-\mu }{\sigma }$$

where *y, μ, σ* represents the normalized value of the x actual value, the mean of feature *x* belongs to, and the feature’s standard deviation.

### 2.3. Ensemble Learning Models

**Figure 2:**
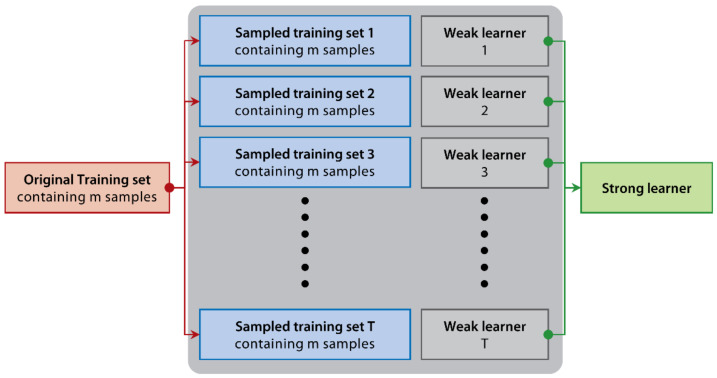
The principle schematic of bagging.

**Figure 3:**
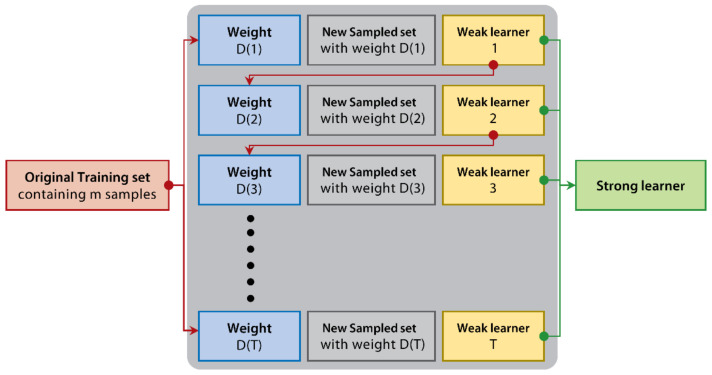
The principle schematic of boosting.

Typically, ensemble learning models have many merits, such as overfitting avoidance, computational advantage, and representation [58,59]. These unique attributes make ensemble learning models the state-of-the-art approach for solving a plethora of machine learning problems [60]. Ensemble learning establishes and combines multiple base learners to achieve significantly superior generalization performance over a single learner. Popular ensemble learning models could be basically categorized into two types: bagging [61] and boosting [62]. The main difference between them is the way to reconstruct train sets and organized base learners. The bagging-based model’s base learners are independent with each other (see Figure 2). The boosting-based model is the opposite, which means that the base learners generated in the previous iterations will guide the next base learner’s learning (see Figure 3). Bagging-based algorithms use repeated sampling with replacements from the original training set to form the new one (see Figure 2). In contrast, boosting-based algorithms assign weights to each sample in the original training set to construct a new one (see Figure 3). Random Forest [63] is a representative bagging-based model. By contrast, XGBoost [64], LightGBM [65], and CatBoost [66] are three successful and popular boosting-based models. All of these four ensemble learning models regard classification and regression trees (CARTs) as base learners.

#### 2.3.1. Random Forest

Random forest [63] is a classical resemble learning model proposed by Breiman et al. in 2001. Each tree in a random forest is built from sampled features extracted from the sampled training set (i.e., bootstrap samples). In addition, instead of using all the features, a subset of the features is randomly selected to achieve the randomization of trees further. Therefore, the bias generated by the random forest increases slightly, but the estimated variance is reduced by the calculated mean value of less-correlated trees, resulting in a better overall performance of the model.

#### 2.3.2. XGBoost

Based on gradient boosted decision tree (GBDT) [64], XGBoost [19] is an ensemble learning algorithm proposed by Chen et al. in 2014. When it comes to XGBoost, GBDT should be briefly introduced at first. GBDT is a successful implementation of boosting, which regards CARTs as base learners. It constructs CARTs iteratively by using the boosting decision tree algorithm, that is, fitting the residual error generated by the previous CARTs. Different from simple regression models, GBDT takes the average of different hypotheses (i.e., a possible relationship between independent and dependent variables) made by base learners to avoid learning a spurious relationship between variables. Moreover, to speed up the convergence, GBDT uses gradient descent to optimize the loss function—mean-square error (MSE) and Huber, for example.

XGBoost is an improved algorithm of GBDT, and the main improvements are as fol-lows. First, the loss function of XGBoost involves second-order Taylor expansion. It makes XGBoost estimate the actual loss function more accurately than that of GBDT, which only considers the first order. Second, XGBoost adds regularizers to depress the overfitting.

#### 2.3.3. LightGBM

LightGBM [65], proposed by Microsoft in 2016, is also an improved algorithm of GBDT. Compared to XGBoost, LightGBM presents gradient-based one-side sampling (GOSS) and exclusive feature bundling (EFB) to speed up the training process. The former can decrease the time cost of calculating loss function, and the latter is aimed at reducing the dimension of samples to accelerate the algorithm. In conclusion, LightGBM can keep almost the same accuracy with less time and space in the setting of big data.

#### 2.3.4. CatBoost

CatBoost [66] is another improved algorithm of GBDT. It was proposed by Yandex in 2017 and its main improvement is in the processing of classification features. CatBoost successfully handles classification features and takes advantage of dealing with them during training rather than preprocessing. Meanwhile, to accelerate the search for the best split, this model uses a kind of histogram computation without any atomic operations, which is also an improvement to LightGBM in terms of time efficiency.

#### 2.3.5. Comparison of the Ensemble Learning Models

XGBoost is better than the other three and the reasons are as follows. In random forest, the training sets of every base learner are generated by repeated sampling with replacements from the original training set. Although they have the same number of samples, the training sets may not include every sample from the original training set. Especially, there are a few real samples in the augmented samples. Therefore, if sampled training sets exclude some real samples, the performance of base learners would acquire degradation. However, XGBoost, based on the boosting framework, does not have this shortcoming. Furthermore, when a CART grows, XGBoost uses the exact greedy algorithm to find the best node. Whereas, LightGBM and CatBoost sacrifice a little precision in finding the best splitting points to improve computational speed and memory usage efficiency, which also adds the model complexity. Although it is feasible and effective in big data, this sacrifice is magnified and decreases the model performance when dealing with small sample problems.

### 2.4. The Data Augmentation

#### 2.4.1. Cubic Spline Interpolation

Zhao found that the cubic spline interpolation is the most potent interpolation for forecasting the direct economic losses of marine disasters among the four interpolations tested [18]. Therefore, we chose the cubic spline interpolation as one of the experiment baselines.

Suppose that there are the following points: $\left(x_{1},\;y_{1}\right)$*,*$\;(x_{2},\;y_{2})\ldots (x_{n},\;y_{n})$ and $a<x_{1}<x_{2}<\cdots <x_{n}<b$. Given n points, the spline curve *S(x)* is a piecewise function. Specifically, the cubic spline equation satisfies the following three conditions:

-  In each segmented section $\left[x_{i},\;x_{i+1}\right]\;\left(i\;=\;1,\;2,\;\ldots ,\;n-1\right)$,$\;S\left(x\right)=S_{i}\left(x\right)$ is a cubic polynomial;
-  $\;S_{i}\left(x\right)=y_{i}\;\left(i\;=\;0,\;1,\;\ldots ,\;n\;\right)$;
-  In section [$a,\;b],\;S\left(x\right)$, derivative $S^{\prime }\left(x\right)$, and second derivative $S’’\left(x\right)$ are all continuous.

Hence,$\;S_{i}\left(x\right)$ can be expressed as follows:

(2) $$\;S_{i}\left(x\right)=a_{i}+b_{i}\left(x-x_{i}\right)+c_{i}{\left(x-x_{i}\right)}^{2}+d_{i}{\left(x-x_{i}\right)}^{3},\;i\;=\;0,\;1,\;\ldots ,\;n-1$$

After substituting the point $\left(x_{i},\;y_{i}\right)$ and the specified first endpoint condition a, b into the matrix equation, we solve the matrix equation and obtain the quadratic differential value $m_{i}$. $a_{i},b_{i},c_{i},d_{i}$ can be calculated as

(3) $$a_{i}=y_{i}$$

(4) $$b_{i}=\frac{y_{i+1}-y_{i}}{x_{i+1}-x_{i}}-\frac{x_{i+1}-x_{i}}{2}m_{i}-\frac{x_{i+1}-x_{i}}{6}\left(m_{i+1}-m_{i}\right)$$

(5) $$c_{i}=\frac{m_{i}}{2}$$

(6) $$d_{i}=\frac{m_{i+1}-m_{i}}{6\left(x_{i+1}-x_{i}\right)}$$

In our experiment, we regard regional direct economic losses as x and one of the features as y, and do interpolation 16 times for 16 different features.

#### 2.4.2. Noise Injection

Most data augmentation algorithms focus on image data classification problems, but there is a data augmentation technique, noise injection [67], that can be applied to non-image data. It is used in the following way (refer to Equations (7) and (8)).

Assume that a sample *i* can be expressed as $X^{i}=\left(x_{1}^{i},x_{2}^{i},\ldots ,x_{m}^{i}\right)$, where m is the dimension of a sample, and corresponding regional direct economic loss is $y^{i}$. For each augmentation, we generate Gaussian noise $\vec{\alpha }=\left(\alpha_{1},\alpha_{2},\ldots ,\alpha_{m}\right)$ and $\alpha_{j}\sim N\left(0,\delta \right)$, then the new sample can be calculated as:

(7) $$X_{new}^{i}=X^{i}+\vec{\alpha }\circ X^{i}$$

(8) $$y_{new}^{i}=y^{i}$$

where ∘ is the symbol of Hadamard product, and according to the convention of unchanging labels on image data, we do not add noise to regional direct economic loss.

#### 2.4.3. KNN-GN

**Figure 4:**
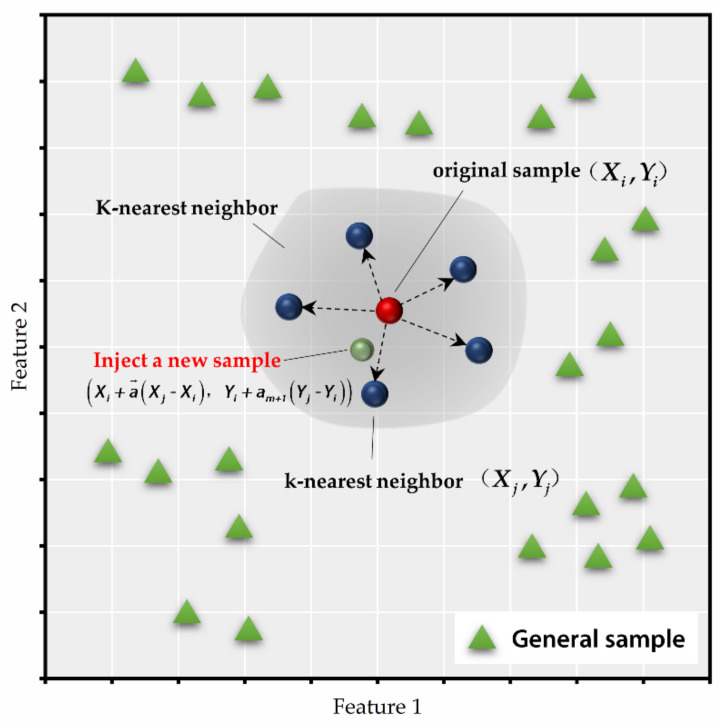
This is the schematic of k-nearest neighbor-Gaussian noise (KNN-GN).

KNN-GN is motivated by noise injection and synthetic minority over-sampling technique (SMOTE), which utilizes the information of neighbors to guide the direction of Gaussian noise (refer to Equations (9) and (10) and Figure 4). New samples can be gained in the following way.

Assume that $\left(X^{j},y^{j}\right)$ is one of the k-nearest neighbors of $X^{i}$ in the (*m*+1)-dimension feature space, we generate an extra random number $\alpha_{m+1}\sim N\left(0,\delta \right)$, then a new sample can be calculated as:

(9) $$X_{new}^{i}=X^{i}+\vec{\alpha }\circ \left(X^{j}-X^{i}\right)$$

(10) $$y_{new}^{i}=y^{i}+\alpha_{m+1}\cdot \left(y^{j}-y^{i}\right)$$

#### 2.4.4. Comparison of the Data Augmentation Technology

KNN-GN is the best of all the data augmentation techniques mentioned above and the reasons are as follows. Cubic spline interpolation is commonly implemented in low-dimensional space. Each feature of new samples calculated by interpolation only establishes a functional relationship with regional direct economic losses, which does not make full use of samples’ high-dimensional characteristics. Moreover, cubic spline interpolation is deterministic and fails to reflect the randomness of the disasters’ process, while noise injection introduces random noise following Gaussian distribution. However, it has its limitations. Noise injection only runs in the feature space, and a new sample is just related to one specific original sample, ignoring the information between the samples. Whereas KNN-GN not only looks for the neighbors in the feature-target space but also adds random directional noise. These merits make full use of samples’ high-dimensional characteristics and the information between samples, as well as introduce randomness, which makes it outperform the former two.

**Figure 5:**
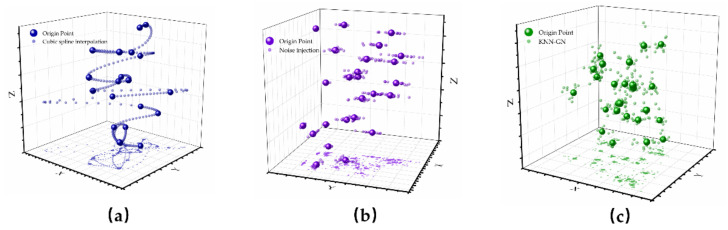
There are the visualizations of these three data augmentation techniques. (**a**)The visualization of cubic spline interpolation; (**b**) The visualization of noise injection; (**c**) The visualization of KNN-GN.

To show the effect of cubic spline interpolation, noise injection, and KNN-GN more intuitively, we apply these techniques to 20 three-dimension samples. The visualizations of the 100-time augmentation are displayed in Figure 5.

### 2.5. Comparative Experimental Design

**Figure 6:**
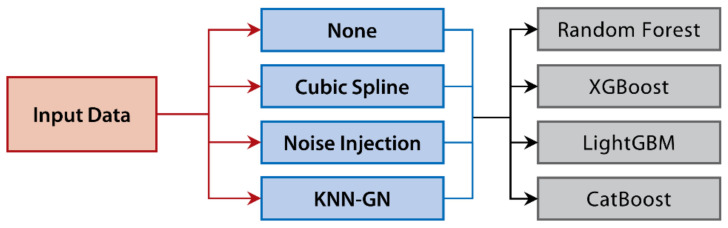
The comparative experiment design of the research.

Due to the advantages mentioned above of KNN-GN and XGBoost, we compared the KNN-GN-based XGBoost method with 15 other data augmentation combined regression models to verify its performance. The baselines of KNN-GN are none augmentation, cubic spline, and noise injection [67], while that of XGBoost are random forest [63], LightGBM [65], and CatBoost [66] (refer to Figure 6). It should be noted that for the sake of convenience, we regarded the none data augmentation and noise injection as kinds of general data augmentation techniques. The original dataset was randomly shuffled and the last five samples were regard as validation samples. We term such a process as one partition of the dataset. To eliminate the randomness while splitting the training and validation set, we adopted ten independently random partitions on the original dataset to generate ten groups of training and validation sets. Meanwhile, to assess the performances of the different combinations, we used MAPE as the error measure, which is defined as follows:

(11) $$e\left(i\right)=pred\left(i\right)-y\left(i\right)$$

(12) $$MAPE=\;\frac{1}{N}\overset{N}{\underset{i=1}{\sum }}\left|\frac{e\left(i\right)}{y\left(i\right)}\right|$$

**Figure 7:**
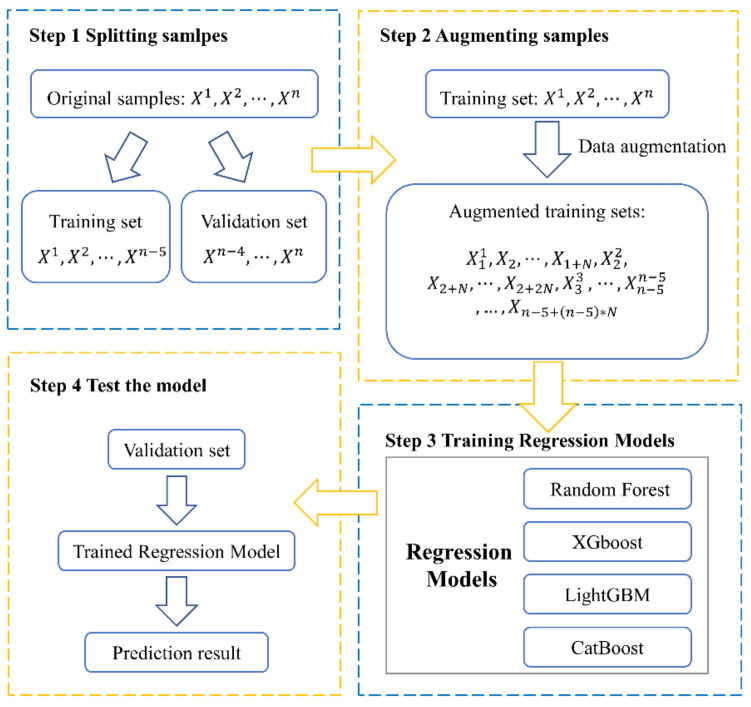
The flow chart of a regression model based on a data augmentation technique.

The flow chart of the experiment in this research is composed of four steps (see Figure 7).

(a) The original samples are randomly split into a training set and a validation set.
(b) According to an acknowledged kind of technique, training sets are augmented with *N* times to attain an extended training set. (The augmented training set by noise injection and KNN-GN includes $\left(N+1\right)\times \left(n-5\right)$ samples, while the augmented training set with cubic spline interpolation and without any data augmentation has $N\times \left(n-5\right)$ samples and n − 5 samples, respectively.)
(c) Augmented training set is fed to one of the regression models.
(d) The validation set is predicted with the trained regression model.

Finally, in addition to comparing the KNN-GN-based XGBoost with other combined models, we compared the model with two widely used models: BPNN and SVR, to further verify its performance.

### 2.6. Parameter Settings

The standard deviations used in noise injection and KNN-GN were respectively set to 0.05 and 0.25, and expansion multiples of both were 10. All baselines were initialized with default parameters suggested by their papers, and we also further carefully adjusted parameters to achieve optimal performance. Especially, for the common parameters among the four models, max_depth and n_estimator were searched in $\left\{2,5,10,15,20\right\}$ and $\left\{16,\;32,\;64,\;128,\;256,\;512,\;1024,\;2048\right\}$. Besides, for XGBoost, we sought the best learning_rate in $\left\{0.001,\;0.01,\;0.1,\;0.2\right\}$.

BPNN had one input layer, two hidden layers, and one output layer with the activation function Sigmoid. SVR used Gaussian kernel and set the other parameters to the default values in the Python library sklearn.

## 3. Results and Discussion

### 3.1. Optimization of Combined Models

**Figure 8:**
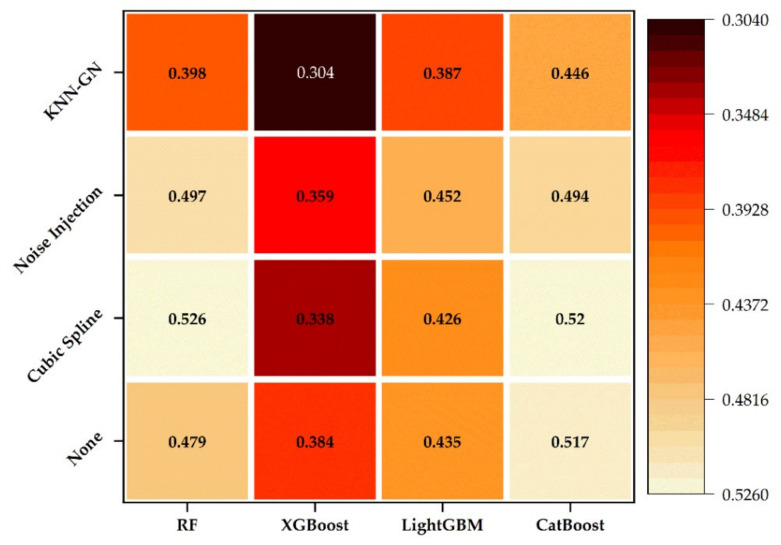
The results of 16 different combined experiments. RF: random forest.

The experimental results of all the combinations are shown in Figure 8. Considering reliability, the results in Figure 8 are the means of 10 repeated experiments using ten different groups of training and validation sets. Moreover, each column represents a regression model, and each row represents a kind of data augmentation technique. The purpose of this experiment is to validate that KNN-GN-based XGBoost is the optimal combined model. Additionally, in Figure 8, the smaller the MAPE value and the darker the color, the better the model performance.

**Figure 9:**
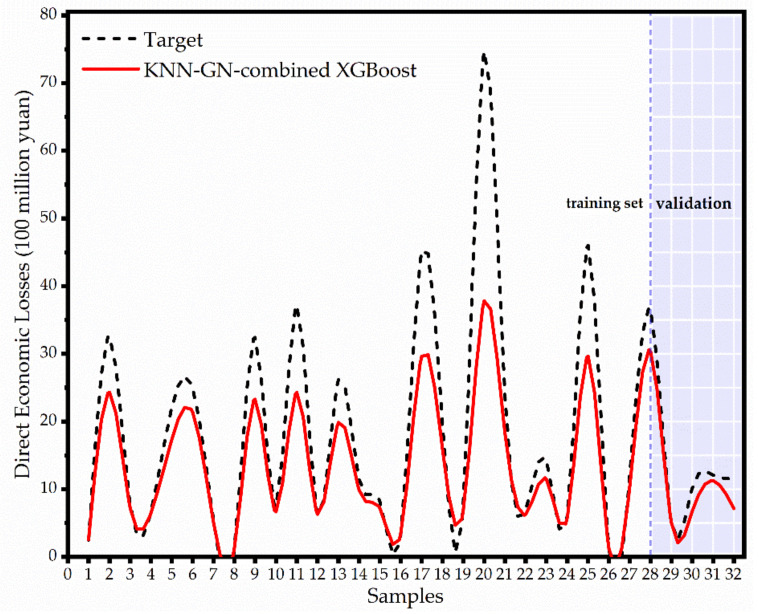
The fitting and prediction result of the KNN-GN-based XGBoost model. The samples in the shadow part belong to validation, while the rest belong to the training set.

In the heatmap, it can be concluded that (1) KNN-GN, on average, performs 27.1%, 18.5%, and 30.0%, respectively, better than none, cubic spline and noise injection on four ensemble learning models, and (2) XGBoost, on average, performs 15.4%, 15.1%, and 14.7% respectively better than random forest, LightGBM and CatBoost on four data augmentation techniques, and (3) KNN-GN-based XGBoost arrives at 0.304 in terms of MAPE and performs best among all the combinations. Figure 9, for example, intuitively shows the KNN-GN-based XGBoost has good prediction performance.

**Figure 10:**
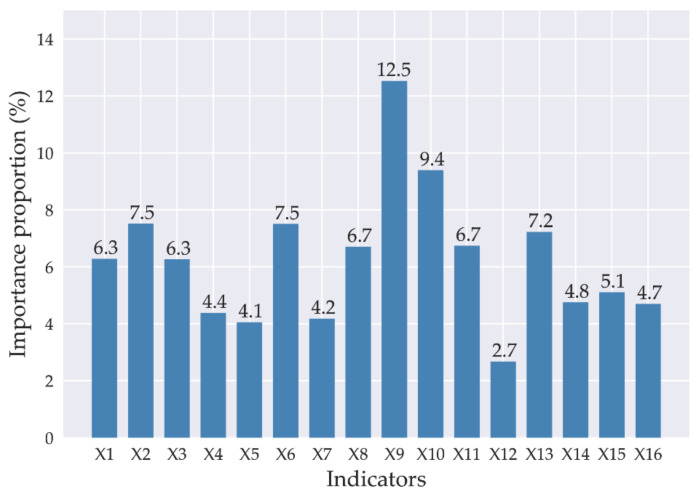
The proportion of the indicator importance based on KNN-GN-based XGBoost.

Therefore, we investigated that KNN-GN-based XGBoost could improve the performance of forecasting regional direct economic losses of storm surge disasters. Furthermore, we also provided the proportion of the indicator importance as shown in the Figure 10. It is observed that the top three most influential, in that order, are the disaster-affected population, the length of marine engineering damage, and the exceeding the local warning water level. The indicator that has the least effect is the unemployment rate.

**Figure 11:**
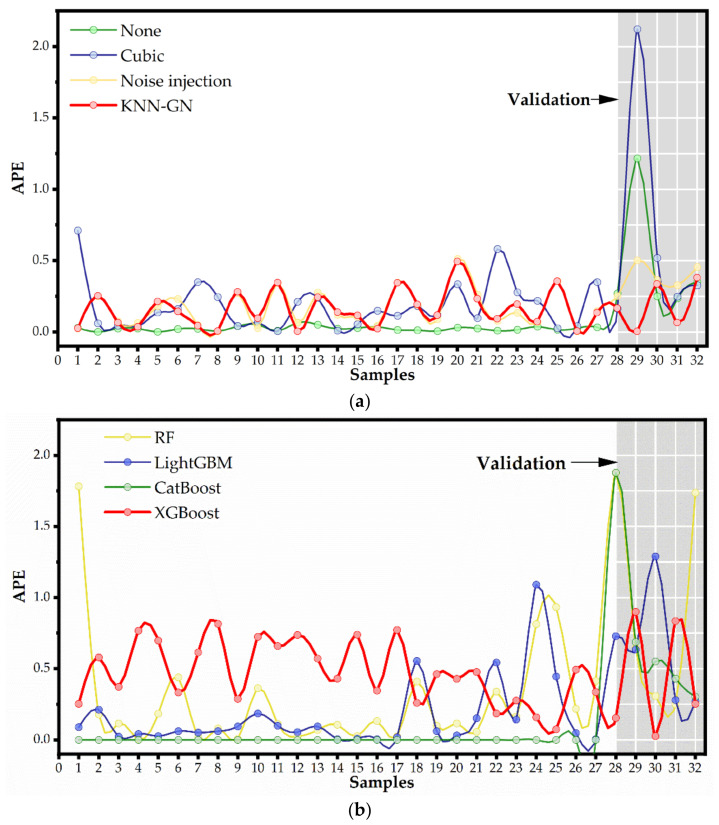
The APE of every sample in one of the partitions. (**a**) The fitting and prediction effect of none data augmentation, cubic spline, noise injection, and KNN-GN based on XGBoost. (**b**) The fitting and prediction effect of random forest, LightGBM, CatBoost, and XGBoost.

**Figure 12:**
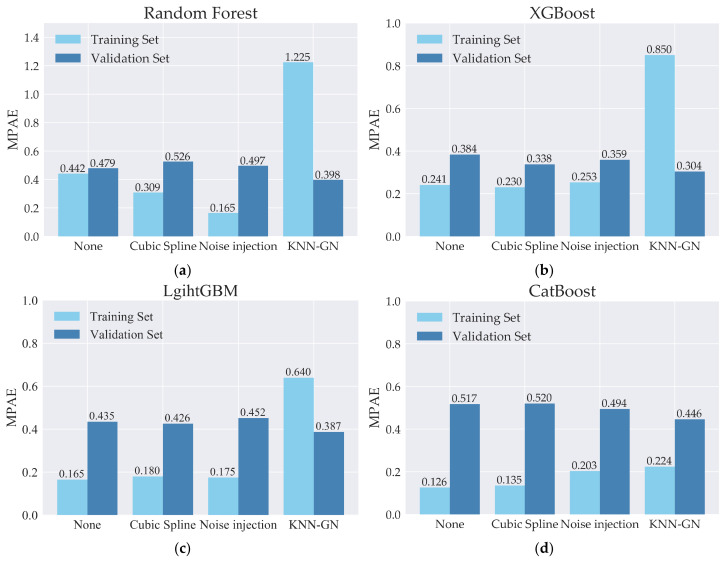
Comparison of MAPE in training and validation sets. (**a**) MAPE of training and validation sets based on random forest. (**b**) MAPE of training and validation sets based on XGBoost. (**c**) MAPE of training and validation sets based on LightGBM. (**d**) MAPE of training and validation sets based on CatBoost.

To explore why KNN-GN-based XGBoost performs best, we made further analysis (see Figure 11 and Figure 12) as follows.

Firstly, to intuitively illustrate the merits of KNN-GN and XGBoost, we show the absolute percentage error (APE) of every sample in one of the partitions (see Figure 11). On the one hand, from Figure 11a, it can be observed that (1) based on XGBoost, none data augmentation has around zero APEs on the training set; besides, KNN-GN, cubic spline, and noise injection have larger APEs on the training set; (2) based on XGBoost, KNN-GN and noise injection have smaller APEs on the validation set than none data augmentation techniques. According to these two observations, we can conclude that KNN-GN and noise injection can suppress the overfitting to promote the model training. On the other hand, Figure 11b shows that (1) random forest has small APEs with high variance on the training set and performs worst on the validation set; (2) LightGBM and CatBoost have lower APEs but perform poorly on the validation set; (3) XGBoost has consistent performance on the training and verification set. The findings above verify that (1) random forest has lousy performance because it omits some real samples as mentioned above, (2) and XGBoost has a better matching degree between the complexity of models and the size of samples.

Furthermore, from Figure 12a, it can be seen that random forest cannot fit in the training set well according to high MAPE at 0.442 and 0.479 respectively on training and validation sets without any data augmentation. Hence, random forest is not a suitable regression model for forecasting regional direct economic losses of storm surge disasters, as analyzed above. Secondly, from Figure 12b–d, it can be seen that the MAPEs of training sets on XGBoost are more extensive than that of LightGBM and CatBoost, and the situation for the validation sets is the opposite. Both observations over again support the opinion that XGBoost is better than LightGBM and CatBoost. Based on the theoretical analysis mentioned above, we can attribute this result to the matching degree between the complexity of models and the size of samples. The higher the matching degree of the model complexity and the sample size, the better model performs. Although LightGBM and CatBoost are the improved models of XGBoost in the setting of big data, XGBoost outperforms the other two in the prediction problem with small samples.

All of the regression models aim at finding a distribution to fit the actual distribution as much as possible by known samples, which is the essence of the model training. However, the scarcity of known samples probably leads to the overfitting of the model. It is because of the mismatch between the complexity of models and the size of samples. Additionally, models are too complicated to learn the unique and individual characteristic of known samples and regard it as the general characteristic of potential samples, thus leading to generalization error and overfitting. This is why we attempt to apply data augmentation to ensemble learning models.

As for the reason that we choose KNN-GN, from Figure 11, it can be seen that (a) based on random forest, KNN-GN performs best for the highest MAPE in training sets; (b) for XGBoost, LightGBM, and CatBoost, MAPEs of training sets appear the increasing trend in the order of none, cubic spline, noise injection and KNN-GN and the situation for validation sets is inverse, which strongly supports that noise injection and KNN-GN could reduce overfitting and further improve the effect of model training; (c) cubic spline interpolation performs worst, even worse than none augmentation data. (d) KNN-GN performs better than the other data augmentation techniques we used as baselines.

Furthermore, according to the observations above, we make an extra theoretical interpretation as follows. The possible reason that cubic spline interpolation performs badly is that it cannot be approximated directly by fitting a simple function due to the complicated relationship between the disaster-causing factor and the regional direct economic loss. Especially when there is a significant difference between the new sample and the original sample, that is, an Euclidean distance of two samples in the feature space. It probably causes the noise introduced by interpolation to be much larger than the information it brings. We try to set new samples as close as the original samples, but not too close to obtain further information. This problem can be tackled by introducing the high dimension Gaussian noise with a small standard as noise injection, and KNN-GN does.

**Figure 13:**
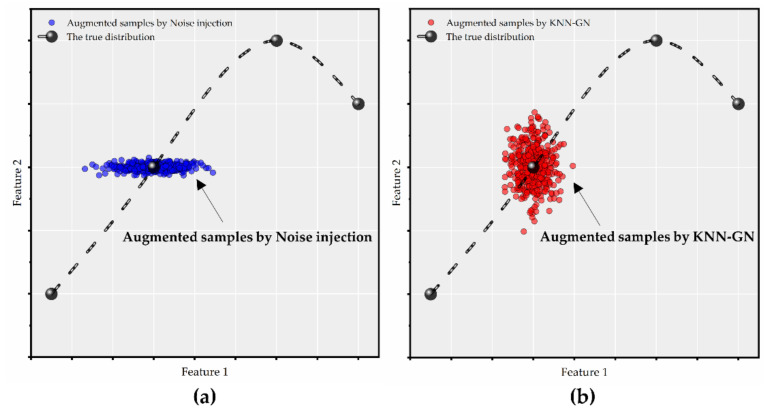
These are the abridged general view to interpret why KNN-GN performs better than noise injection. (**a**) The effect picture of noise injection. The blue part includes points augmented by noise injection. According to the application on image data, noise injection only adds noise to the Feature 1 dimension. (**b**) The effect picture of KNN-GN, which adds noise to two dimensions and tends to be close to the true distribution. The yellow part includes points augmented by KNN-GN.

Figure 11 explains the reason why KNN-GN performs better than noise injection. Suppose we have a class of samples with two-dimension points. The actual distribution of these samples and points is shown as the dotted line in Figure 13a,b. Samples augmented by noise injection and KNN-GN are shown in Figure 13a,b. Intuitively, samples generated by KNN-GN are easier to hit the true curve so that more information can be brought to guide to fit the real distribution. Because when expanding a sample, noise injection only expands a sample approximately in its own feature vector direction, KNN-GN does more. It first finds the sample’s neighbor in the features-target space and then introduces a Gaussian noise tending to the difference in vector direction between the sample and its neighbor. From the perspective of mathematics, the fundamental idea of KNN-GN is like using the secant line instead of the tangent line and further replacing the curve.

Therefore, we can finally conclude that KNN-GN-based XGBoost improves the performance of storm surge disasters’ regional direct economic losses evaluation by applying XGBoost with suitable model complexity and suppressing the overfitting of model training.

### 3.2. Validation of the KNN-GN-Based XGBoost Model

We compared the KNN-GN-based XGBoost with XGBoost and two widely-used models: BPNN and SVR to assess the optimized ensemble learning model’s performance. For the models being compared, we used the dataset of Fujian and obtained ten different partitions to eliminate contingency. Same as the previous experiment, we used MAPE to evaluate the performances of the models. The experimental results of the different models are listed in Table 2. When the MAPE of validation is lower, the performance is better.

From Table 2, we can observe that the proposed optimized model has the minimum MAPE of validation sets. On the one hand, for the reason that KNN-GN-based XGBoost performs better than BPNN and SVR, KNN-GN-based XGBoost adopted KNN-GN to solve the data scarcity problem and eventually depressed the overfitting of XGBoost. On the other hand, the reason that KNN-GN-based XGBoost outperforms BPNN and SVR is as follows. When dealing with small sample problems, the general learning algorithm can often find many different hypotheses that fit the training samples well. However, it is still difficult to make accurate predictions for unknown samples, which is overfitting. There is serious overfitting in Table 2, where BPNN and SVR have low MAPEs (<0.2) in the training sets but perform poorly (MAPE > 0.8) in the validation sets. However, ensemble learning models, such as XGBoost and KNN-GN-based XGBoost, select the average of different hypotheses to reduce overfitting and improve overall generalization ability. We can therefore conclude that the novel approach is effective and could improve the forecasting performance.

### 3.3. Robustness Analysis

To assess the robustness of the KNN-GN-based XGBoost, we applied it to another dataset that includes 35 storm surge disaster samples from the years 1995–2019 in Guangdong. We selected the 30 storm surge samples that occurred between 1995 and 2015 as the training set. The disaster samples from 2016 to 2019 were the verification samples (the last five samples). It should be noted that we have appropriately adjusted the robustness experiment models’ parameters to be more suitable for the Guangdong dataset.

**Figure 14:**
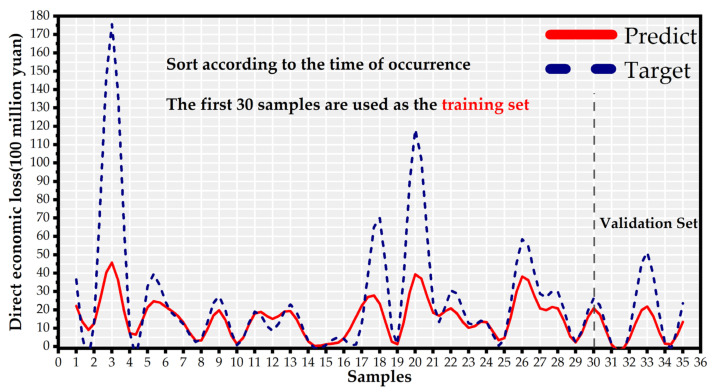
The results of the robustness experiment of Guangdong.

The fitting-prediction values and targets of samples are shown in Figure 14, where we can observe that KNN-GN-based XGBoost has a considerable good prediction on the validation set. Although the proposed model did not predict the regional direct economic losses of 33rd and 35th storm surge disasters so well, the storm surge disaster itself has uncertainty and randomness. Therefore, we can conclude that KNN-GN-based XGBoost has robustness. This model’s performance is qualified to aid policy makers in taking timely and proper measures in managing a storm surge disaster.

## 4. Conclusions

This paper proposed a novel evaluation system consisting of an index system and a regression model, which could accurately predict regional direct economic losses after storm surge disasters in a short time. The performance is superior to that of state-of-the-art models from two advantages: (1) the index system considers four aspects: disaster-causing factors, disaster-formative environments, hazard-bearing bodies, and disaster prevention capabilities from the perspectives of storm surge disasters’ formation mechanism and risk management; (2) the KNN-GN-based XGBoost regression model not only reduces the overfitting by taking the average of hypotheses and data augmentation but also matches the model complexity and the sample size well.

Our evaluation model achieves promising results on two storm surge disaster datasets in the Fujian and Guangdong provinces in China. It is worth noting that the performance still has room for improvement by training on more datasets. As for the limitation, the 16 indicators selected in the index system of this paper are mainly from historical data. In the future, as the storm surge forecasting systems and the numerical simulation technology advance, these indicators could be estimated accurately before a disaster happens. This progress makes the proposed evaluation system work even before the storm surge disaster happens. Finally, the results show that our evaluation system can be applied to other disasters—geography-related disasters, for instance—with small samples.

## Figures and Tables

**Table 1:** The storm surge disaster loss assessment index system.

Criteria	Indicators	Variable	References
Disaster-causing factors	Maximum storm surge (cm)	$X_{1}$	Guo 2020 [25], Shi 2020 [23], Wang 2018 [9], Yang 2016 [22], Nicholls 2008 [33]
	Exceeding the local warning water level (cm)	$X_{2}$	Guo 2020 [25], Shi 2020 [23], Wang 2018 [9], Yang 2016 [22]
	Duration of the typhoon (hour)	$X_{3}$	Guo 2020 [25]
Disaster-formative environment	Urban green area (hm^2^)	$X_{4}$	Sun 2020 [24], Stefanidis 2013 [26], Peduzzi 2009 [34], Pelling 2004 [35]
	The sown area of crops (hm^2^)	$X_{5}$	Guo 2020 [25], Stefanidis 2013, Peduzzi 2009 [34], Pelling 2004 [35]
	Aquaculture area (hm^2^)	$X_{6}$	Guo 2020 [25], Nicholls 2008 [33]
	The proportion of the old and young population (%)	$X_{7}$	Sun 2020 [24], Koks 2015 [27], Cutter 2000 [30]
	The proportion of the urban population (%)	$X_{8}$	Guo 2020 [25], Sun 2020 [24], Almeida 2016 [31], Peduzzi 2009 [34], Pelling 2004 [35]
Disaster-affected bodies	The disaster-affected population (10,000)	$X_{9}$	Dickson 2012 [36], Wang 2018 [9]
	The length of marine engineering damage (km)	$X_{10}$	Wang 2018 [9], Lam 2017 [37]
Disaster prevention capabilities	GDP per capita (CNY 1)	$X_{11}$	Guo 2020 [25], Sun 2020 [24], Wang 2018 [9], Almeida 2016 [31], Peduzzi 2009 [34], Nicholls2008 [33], Pelling 2004 [35], Davidson 2001 [28]
	The unemployment rate (%)	$X_{12}$	Sun 2020 [24], Peduzzi 2009 [34], Pelling 2004 [35]
	Fiscal expenditure (CNY 100 M)	$X_{13}$	Sun 2020 [24], Ainuddin 2015 [32], Cardona 2006 [29]
	The number of beds per thousand people	$X_{14}$	Guo 2020 [25], Sun 2020 [24], Wang 2018 [9], Almeida 2016, Ainuddin 2015 [32], Davidson 2001 [9]
	Number of medical institutions	$X_{15}$	Guo 2020 [25], Sun 2020 [24], Wang 2018 [9], Ainuddin 2015 [32]
	Commercial insurance costs (CNY 100 M)	$X_{16}$	Guo 2020 [25], Ainuddin 2015 [32], Cardona 2006 [29]

**Table 2:** The MAPE of training and validation sets in the different models.

	KNN-GN-Based XGBoost	XGBoost	BPNN	SVR
Training set	0.450	0.241	0.175	0.029
Validation set	0.304	0.384	0.857	0.837

## Data Availability

Data sharing not applicable. No new data were created or analyzed in this study. Data sharing is not applicable to this article.
